# Deep Learning to Distinguish Edema Secondary to Retinal Vein Occlusion and Diabetic Macular Edema: A Multimodal Approach Using OCT and Infrared Imaging

**DOI:** 10.3390/jcm14031008

**Published:** 2025-02-05

**Authors:** Guilherme Barbosa, Eduardo Carvalho, Ana Guerra, Sónia Torres-Costa, Nilza Ramião, Marco L. P. Parente, Manuel Falcão

**Affiliations:** 1 INEGI—Instituto de Ciência e Inovação em Engenharia Mecânica e Engenharia Industrial, 4200-465 Porto, Portugal; aguerra@inegi.up.pt (A.G.); nramiao@inegi.up.pt (N.R.); 2 Department of Ophthalmology, CHUSJ—Centro Hospitalar e Universitário de São João, 4200-319 Porto, Portugal; sonia.torres.costa@gmail.com (S.T.-C.); falcao@med.up.pt (M.F.); 3 Department of Mechanical Engineering, Faculdade de Engenharia, Universidade do Porto, 4200-465 Porto, Portugal; mparente@fe.up.pt; 4 Department of Surgery and Physiology, Faculty of Medicine, University of Porto, 4200-319 Porto, Portugal

**Keywords:** deep learning, optical coherence tomography, retinal vein occlusion, diabetic macular edema, convolutional neural network

## Abstract

**Background:** Retinal diseases are emerging as a significant health concern, making early detection and prompt treatment crucial to prevent visual impairment. Optical coherence tomography (OCT) is the preferred imaging modality for non-invasive diagnosis. Both diabetic macular edema (DME) and macular edema secondary to retinal vein occlusion (RVO) present an increase in retinal thickness, posing etiologic diagnostic challenges for non-specialists in retinal diseases. The lack of research on deep learning classification of macular edema secondary to RVO using OCT images motivated us to propose a convolutional neural network model for this task. **Methods:** The VGG-19 network was fine-tuned with a public dataset to classify OCT images. This network was then used to develop three models: unimodal—the input is only the OCT B-scan; multimodal—the inputs are the OCT B-scan and diabetes information, and multi-image—the inputs are the OCT B-scan, the infrared image, and the diabetes information. Seven hundred sixty-six patients from ULS São João were selected, comprising 208 healthy eyes, 207 with macular edema secondary to RVO, 218 with DME, and 200 with other pathologies. The performance metrics include accuracy, precision, recall, F_0.5_ score, and area under the receiver operating characteristic curve (AUROC). **Results:** The multi-image model achieved better results, with an accuracy of 95.20%, precision of 95.43%, recall of 95.20%, F_0.5_-score of 95.32%, F_1_-score of 95.21%, and AUROC of 99.59% on the classification task between four classes. **Conclusions:** This study presents a novel method to distinguish macular edema secondary to RVO and DME using diabetes diagnosis, OCT, and infrared images. This research aims to provide a reliable tool for ophthalmologists, improving the accuracy and speed of diagnoses.

## 1. Introduction

Retinal vein occlusion (RVO) is the second most common retinal vascular disorder after diabetic retinopathy and is a significant cause of visual loss and visual disability. RVO occurs due to the obstruction of the retinal venous system by thrombus formation, leading to increased capillary pressure, fluid leakage, and macular edema, which are primary contributors to vision impairment in these patients [1,2].

Approximately 16 million people worldwide are affected by RVO, with its prevalence influenced by factors such as age, gender, and underlying health conditions. The incidence of RVO increases with age, particularly affecting individuals over 60 [3].

RVO is broadly classified into branch RVO (BRVO) and central RVO (CRVO) based on the site of occlusion. BRVO typically occurs at arteriovenous crossings, whereas CRVO occurs at or near the lamina cribrosa of the optic nerve. The compression by adjacent atherosclerotic retinal arteries is the most common etiological factor. CRVO is characterized by disc edema, increased dilatation and tortuosity of retinal veins, widespread hemorrhages, cotton wool spots, macular edema, and capillary non-perfusion across the retina. BRVO exhibits similar features but is confined to the area drained by the affected vein. Visual acuity loss primarily results from macular edema but can also arise from macular ischemia or neovascular complications such as vitreous hemorrhage and neovascular glaucoma [1].

Optical coherence tomography (OCT) is a non-invasive imaging technique that provides detailed cross-sectional images of retinal structures, facilitating the diagnosis of macular edema secondary to RVO, hereafter mentioned solely as RVO. OCT typically uses near-infrared (IR) light, which has a penetration depth of several hundred microns in tissue. The interferometric setup reconstructs the depth profile of the retina, providing high-resolution images. Combining OCT with confocal scanning laser ophthalmoscopy (cSLO) allows for motion tracking and precise re-examination of specific retinal regions. Advances in functional OCT now permit imaging of retinal blood flow, tissue polarization, and mechanical properties such as elasticity. The SPECTRALIS device by Heidelberg Engineering integrates cSLO and OCT, providing versatile imaging capabilities including reflectance imaging in various wavelengths. This system’s motion tracking and high-resolution imaging are particularly beneficial for diagnosing and monitoring RVO. These include cSLO reflectance imaging in the near IR. OCT is usually combined with IR confocal imaging, though other combinations are possible as well. Confocal imaging creates a transversal image of the retina corresponding to the en-face plane of OCT. The SPECTRALIS system utilizes the IR cSLO scans for automatic motion tracking [4].

Computer-assisted diagnosis (CAD) with artificial intelligence (AI) has demonstrated promising diagnostic performance in image recognition, achieving robust results across various medical specialties, including ophthalmology. In particular, AI can be utilized to screen OCT scans for disease detection, helping to mitigate factors such as human bias, fatigue, and mindset [5].

Early approaches to CAD involved the extraction of relevant features, which required domain expertise and varied with the dataset. These features could include texture or structural information of the image. Texture features could be created using techniques such as local binary pattern and filtered using principal component analysis or k-means clustering, although they were more susceptible to noise. Structural features, on the other hand, were more device-independent and thus provided more reliable classifications [6].

More recently, deep learning (DL) models have been employed to classify OCT scans and the development of convolutional neural networks (CNN) has improved the performance of image classification, significantly improving the performance of image classification [7,8].

CNNs utilize convolutional operators to extract features directly from image pixels, making them highly effective for image classification tasks. The images are processed through convolutional, pooling, and activation layers to extract features, and then fully connected layers are used to classify the image [9]. These models have been applied to classify multiple diseases simultaneously, which is more practical for real-world disease screening. For example, Kermany et al. [10] developed a DL model capable of distinguishing eyes with choroidal neovascularization (CNV) or diabetic macular edema (DME) from eyes with drusen or healthy eyes. The authors of this work made the used dataset publicly available, which has been used in further research. One example is the study from Li et al. [11], which achieved excellent results using the VGG16 network with an accuracy of 98.6%.

However, there is a research gap in differentiating RVO from DME. Most published works that address the classification between these two retinal pathologies rely on fundus images. For example, Choi et al. [12] used random forest transfer learning (TL) based on the VGG-19 architecture to classify fundus images between diabetic retinopathy (DR) and RVO, with an accuracy of 74.7%. Abitbol et al. [13] developed a DL model to distinguish widefield color fundus images of patients with RVO and DR, with an accuracy of 85.2% for DR and 88.4% for RVO. Other works focused only on the distinction between different types of RVO and healthy eyes on fundus images [14,15].

Previous studies, such as the one from Li et al. [11], have achieved high accuracy in classifying the OCT images from Kermany dataset [16]. However, these models often fail to incorporate additional patient data, such as diabetes diagnosis, which can significantly enhance classification accuracy. Regarding OCT images, there is limited research on the classification of patients with RVO and this exam is the preferred imaging modality for non-invasive diagnosis. Therefore, the development of a model that classifies RVO with this exam has tremendous potential in clinical applicability.

Pin et al. [17] proposed an ensemble of two TL models, MobileNetV3Large and ResNet50, to classify OCT images between RVO, age-related macular degeneration (AMD), central serous chorioretinopathy (CSCR), and DME with an overall accuracy of 91.69%. Khan et al. [18] modified three pre-trained models and extracted features via TL. The best features were selected using ant colony optimization and passed to the k-nearest neighbors and support vector machine algorithms to classify OCT images between AMD, RVO, CSCR and DME with an accuracy of 99.1% with ant colony optimization and 97.4% without it. Recently, Kulyabin et al. [19] tested the performance of VGG16 and ResNet50 on an open-access dataset [20] of OCT images, achieving an overall accuracy of 89.5% with VGG16. This dataset included AMD, DME, Epiretinal Membrane, Retinal Artery Occlusion, RVO, and Vitreomacular Interface Disease.

This study aims to develop a DL-based method to classify specific retinal conditions involving edema, namely DME, RVO-associated edema, and generic disease, using OCT and infrared imaging.

## 2. Materials and Methods

### 2.1. Ethical Approval

The study was approved by the Institutional Ethics Review Board of ULS São João. The protocol conformed with the canons of the Declaration of Helsinki for research involving human participants, as well as the European Union’s General Data Protection Regulation.

Informed consent was waived in view of the retrospective nature of the study. The dataset includes retinal OCT images, IR images, and patient data such as diabetes status. All personal identifiers were removed to ensure patient anonymity and data confidentiality.

### 2.2. Datasets

The development of this work utilized two datasets: the publicly available Kermany dataset [16], and a private dataset from ULS São João in Porto, Portugal (HSJ dataset). The Kermany dataset [16] comprises four classes: CNV, DME, drusen, and normal. It includes over one hundred thousand OCT images obtained with Heidelberg Spectralis, selected from retrospective cohorts of adult patients from multiple institutions, making it a robust resource for training models to recognize a wide range of structures and forms in OCT scans. However, this dataset contains some duplicate images, which were removed to enhance model performance. Table 1 shows the number of images for each class before and after removing duplicates, along with the number of patients after duplicate removal.

**Figure 1:**
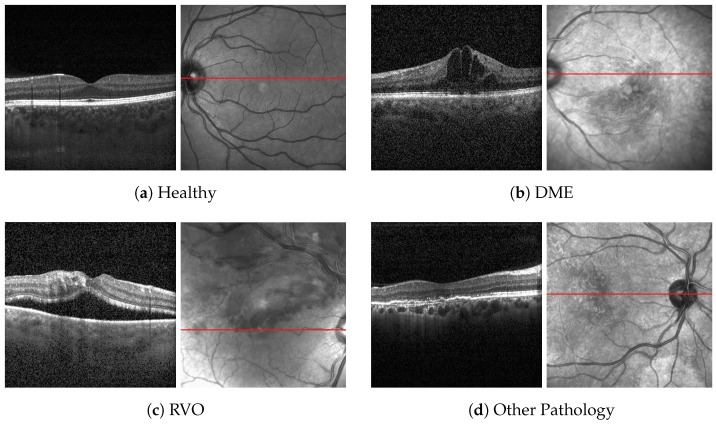
OCT B-scan (**left**) and IR (**right**) images.

Due to the limited number of public datasets with OCT images from RVO patients, a private dataset was created, including OCT scans of healthy eyes and eyes with RVO, DME, and other retinal pathologies. Initially, the dataset comprised three classes: normal, RVO, and DME. To enhance the model, a fourth class was added to include other pathologies apart from DME and RVO. The patients were classified by two ophthalmologists (M.F. and S.C.). An example of OCT scans centered on the fovea and the IR image of each class are shown in Figure 1.

**Figure 2:**
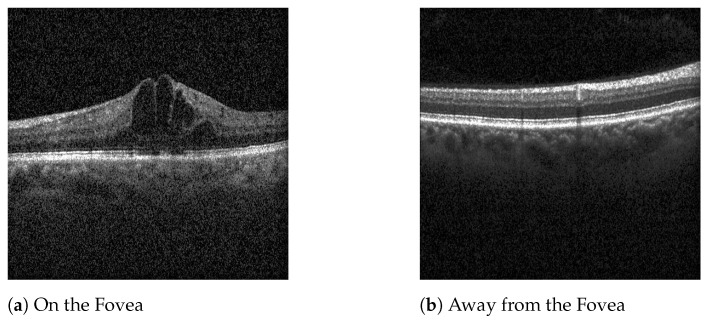
Scans of a patient with DME.

In preliminary work, models trained on all OCT volume scans misclassified images away from the fovea, where pathology signs might be absent. Some examples of patients’ scans with DME, centered on the fovea and further away from it, are shown in Figure 2. Consequently, only the five scans most centered on the fovea were included. Table 2 provides the number of OCT scans, eyes, and patients, along with the distribution of age, sex, and diabetes diagnosis.

### 2.3. Kermany Model

Before supplying the CNNs with images, preprocessing was applied to remove speckle noise and artifacts. Modern OCT machines’ faster scanning speeds can introduce sample-based speckle [21]. Additionally, the public dataset contained rotated images and white spaces that could affect the model performance. The preprocessing for the Kermany dataset included converting white borders to black pixels, applying the morphological top-hat operation to enhance contrast and reduce noise, cropping images to a region of interest based on retinal layers, converting pixels above the retinal nerve fiber layer to black to reduce speckle noise and artifacts, and rescaling images to a uniform size of 224 × 224 pixels.

**Figure 3:**
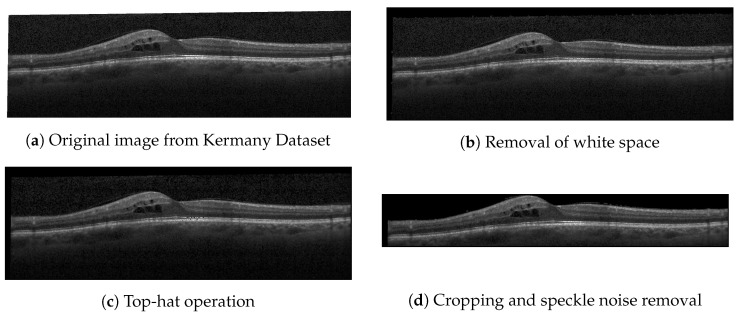
Preprocessing results.

Figure 3 shows the results from the preprocessing applied to the images from the Kermany dataset.

A total of 250 images per class were selected for the testing set, with the remaining images divided into training and validation sets using five-fold cross validation. Table 3 outlines the image distribution across these sets.

To address class imbalance, a weighted random sampler was used during training to ensure equal sampling probability for each class.

The cross-entropy loss function used during training is defined as

(1) $$CE_{Loss}\left(\theta \right)=-\frac{1}{N}\sum_{i=1}^{N}y_{i}\cdot \log\left({\hat{y}}_{i}\left(X_{i},\theta \right)\right),$$

where θ represents the model’s trainable parameters, $X_{i}$ denotes the OCT image, $y_{i}$ is the image label, *N* is the number of training images, and ${\hat{y}}_{i}\left(X_{i},\theta \right)$ is the predicted probability after applying the Softmax function.

To prevent overfitting, L2 regularization was used, and an additional term was considered in the loss function:

(2) $$Loss\left(\theta \right)=CE_{Loss}\left(\theta \right)+\alpha \cdot \theta^{2},$$

where α is the parameter that governs the regularization.

The VGG-19 model [22] was used with batch sizes of 16, 32, and 64. Transfer learning (TL) was employed, and the initializing model weights were those obtained from a pre-trained VGG-19 on the ImageNet dataset [23].

### 2.4. HSJ Model

The HSJ dataset was split using patient-wise separation, ensuring that images from the same patient did not appear in different sets. This approach prevents data leakage and enhances model generalization. Additionally, to generalize the algorithm to both left and right eyes, data augmentation techniques, including image flipping in OCT and IR images, were applied, simulating scans of both eyes within the dataset. Various approaches were tested, starting with a three-class problem (Normal, RVO, and DME) and different inputs: only an OCT scan (unimodal model), an OCT scan with diabetes information (multimodal model), and an OCT scan with diabetes information and an IR image (multi-image model). Subsequently, a four-class problem (Normal, RVO, DME, and Other) was considered, using the best input combinations from the three-class model. In all approaches, the weights of the Kermany Model were used to initialize the weights of the classification model. The diabetic information included in this dataset was limited to a binary classification indicating whether the patient had diabetes or not.

**Figure 4:**
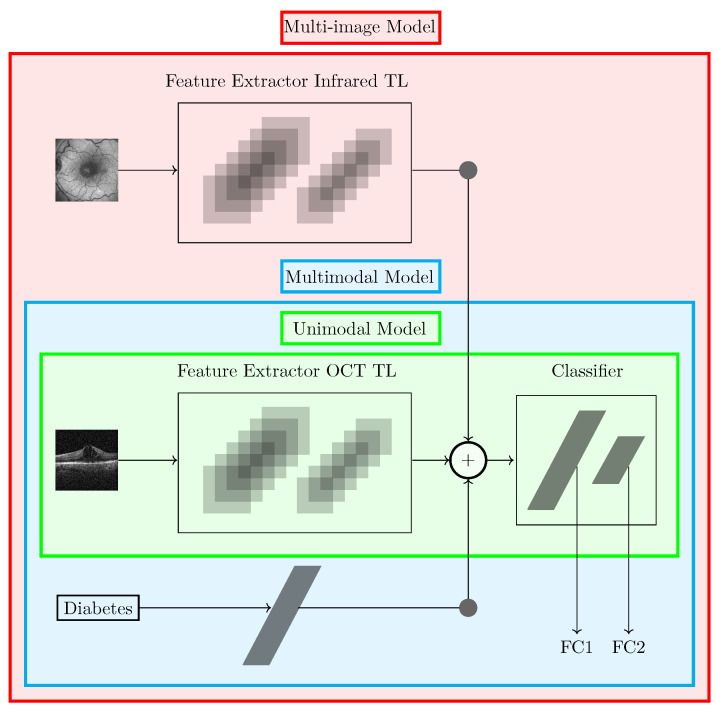
Overview of the proposed models. (TL—transfer learning; FC—fully connected; OCT—optical coherence tomography).

An overview of the proposed models with these 3 different inputs is shown in Figure 4. The green area represents the unimodal model, which has only the OCT image as input. The underlying cyan rectangle represents the multimodal model, which uses information regarding whether or not the patient has diabetes. This input was passed through a fully connected layer and the output is summed to the output of the feature extractor. Finally, the multi-image model corresponds to the red area and it considers an additional input, the IR image. This input is passed through a CNN to extract the features of these images, and the output is summed to the other outputs before going through the fully connected layers of the classifier.

Data augmentation techniques, such as horizontal flip, Gaussian blur with a kernel size of 5 and standard deviation of 0.6, and their combination, were applied on the fly to the training data with a probability of 30% to enhance data diversity and model generalization. It artificially generates more data to train by creating variations of the original images.

During training, a cosine annealing schedule was employed to adjust the learning rate, defined as

(3) $$L_{r_{t}}=L_{r_{min}}+\frac{1}{2}\left(L_{r_{max}}-L_{r_{min}}\right)\left(1+\cos\left(\pi \frac{T_{cur}}{T_{max}}\right)\right),$$

where $L_{r_{t}}$ is the current learning rate, $L_{r_{max}}$ and $L_{r_{min}}$ are the maximum and minimum learning rates, $T_{cur}$ is the current iteration, and $T_{max}$ is the maximum number of iterations.

The dataset was split into training, validation, and testing sets, ensuring no patient overlap between sets. The testing and validation sets contained 5 images of 25 eyes in each class. Table 4 shows the number of images per class for each set, including diabetes status (without diabetes (D-), with diabetes (D+), and without information (D?)).

Different TL strategies were analyzed: feature extractor (only classifier parameters trained), fine-tuning (partially freezing the feature extractor), and fine-tuning from scratch (training all parameters) [24]. The classifier from the pre-trained model has two hidden layers with 4096 neurons; however, for the proposed architecture, only one hidden layer, denoted by FC1 in Figure 4, was used. The last layer of the classifiers, denoted by FC2, is the output layer with 3 or 4 neurons depending on the number of classes of the problem. Table 5 shows the hyperparameters and the values used in the fine-tuning process.

**Figure 5:**
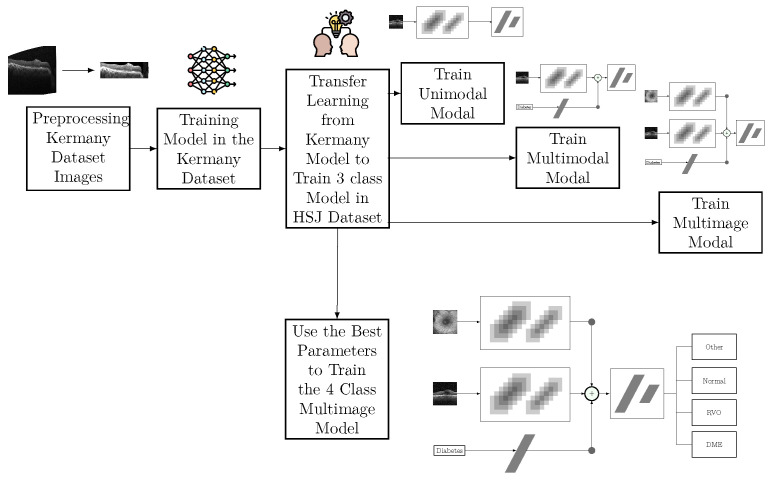
Overview of the developed methodology.

The diagram in Figure 5 presents a summary of the methods in this work.

## 3. Results

All models were trained for a maximum of 100 epochs; Adam optimization was used, and early stopping was applied to avoid overfitting during training by monitoring the validation F_β_-score. If this metric did not increase for 10 epochs, training would stop automatically. The training of the proposed models was performed on an NVIDIA RTX A6000 GPU workstation with 32 or 64 GB of RAM, depending upon availability.

The evaluation metrics considered were the accuracy (A), F_0.5_-score (F_0.5_), precision (P), recall (R), and area under the receiver operating characteristic curve (AUROC). The threshold for the layer decision making was 0.5.

### 3.1. Kermany

**Figure 6:**
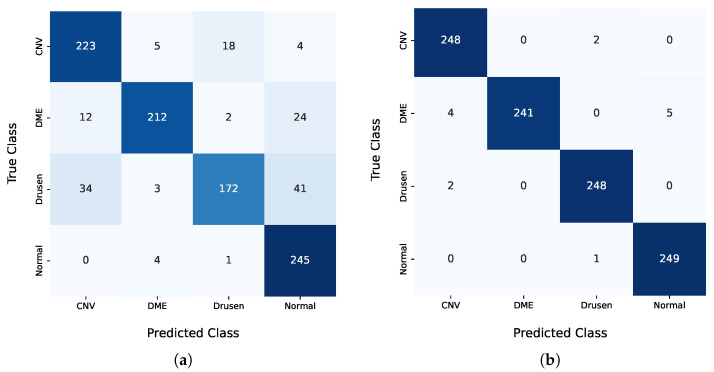
Confusion matrix of models using Kermany dataset. (CNV—choroid neo-vascularization; DME—diabetic macular edema). (**a**) Trained model. (**b**) Model from Li et al. [11].

For the Kermany model, the dropout was maintained at 0.5, and a batch size of 64 and a learning rate of 0.00001 were used. Cross validation was applied, and the performance metrics and confusion matrix from the best fold are shown in Figure 6 and Table 6, respectively. The results obtained by Li et al. [11], the model that achieved the best performance on the Kermany dataset, are also displayed for comparison.

### 3.2. Three-Class Problem

**Figure 7:**
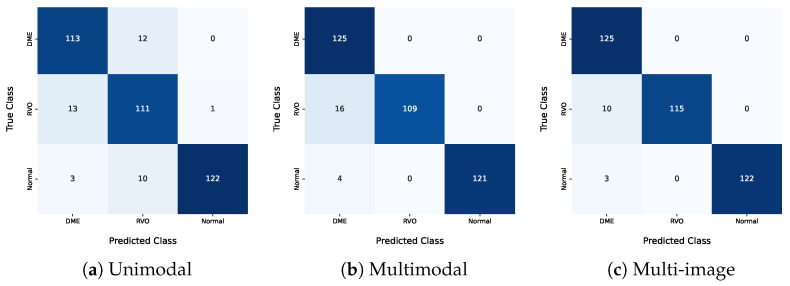
Confusion matrices for the three-class problem. (DME—diabetic macular edema; RVO—retinal vein occlusion).

After a first hyperparameter grid search, it was clear that, of the hyperparameters from Table 5, some had a small influence on the model’s performance. Therefore, the dropout value was fixed at 0.7, the activation function used was the ELU, the maximum number of iterations $T_{\max}$ was fixed at 0, and the number of neurons of FC1 was fixed at 256. Fine-tuning from scratch was applied after initializing the weights with those from the Kermany model, as it yielded the best performance during hyperparameter optimization. The hyperparameters with the most influence were the batch size, the learning rate, α, and the type of data augmentation. As a consequence, a grid search with 54 combinations was performed for each of the proposed architectures, and the mean of the performance metrics with a confidence interval of 95% is shown in Table 7, as well as the metrics of the best model. These models were selected based on the testing and validation F_β_-scores. The respective confusion matrices are shown in Figure 7.

### 3.3. Four-Class Problem

**Figure 8:**
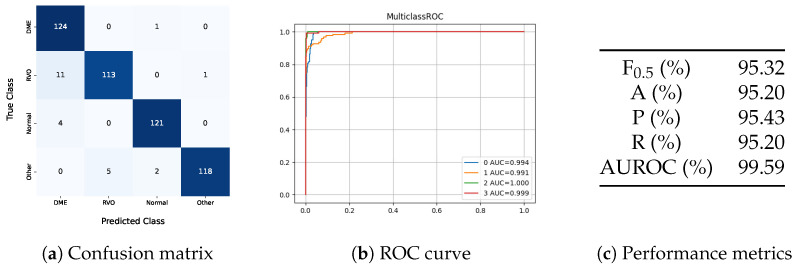
Results of the four-class problem. (DME—diabetic macular edema; RVO—retinal vein occlusion; F_0.5_—F_0.5_ score; A—accuracy; P—precision; R—recall; AUROC—area under the receiver operating characteristic curve).

The architecture that achieved the best results in the three-class problem was adapted for the four-class problem. The optimal model was trained using a batch size of 16, an initial learning rate of 0.0001, and an α of 0.01. Data augmentation techniques, including horizontal flip and Gaussian blur, were employed to enhance the training data. The corresponding confusion matrix, receiver operating characteristic (ROC) curve, and performance metrics are presented in Figure 8.

## 4. Discussion

The use of AI in the diagnosis of retinal diseases can significantly enhance the capabilities of ophthalmologists, enabling them to deliver faster and more accurate diagnoses. This efficiency gain not only improves patient outcomes by allowing earlier detection and treatment of retinal diseases but also increases the number of medical appointments that ophthalmologists can handle, thereby extending their reach and impact.

In the pre-training step, the results obtained with the public dataset from Kermany et al. [16] were significantly below the state-of-the-art results. The F_0.5_ score of 85.52% achieved by the proposed model lagged more than 10 percentage points behind the results from Li et al. [11]. Nonetheless, this model was only used to initialize the weights of the classification model. The decision to use the weights of the Kermany model as a starting point for the multimodal and multi-image models was based on the benefits of TL, even when the pre-trained model was developed using a single modality, in this case, OCT images. TL is a widely adopted approach in DL, where models pre-trained on related tasks provide a better initialization for new tasks, thereby accelerating convergence and potentially improving the model performance. While IR images were not present in the Kermany dataset, the features learned from OCT images contributed to the model’s ability to generalize better during training. Hyperparameter optimization demonstrated that models initialized with Kermany weights and subsequently fine-tuned on our dataset outperformed models trained from scratch. This highlights the importance of leveraging domain-relevant pre-training even when modalities differ slightly. However, the results also underline the need for further research into pre-training strategies that simultaneously address multimodal inputs, as the inclusion of IR images and clinical features poses unique challenges and opportunities for developing robust AI systems.

Regarding the HSJ model used for classification, three different architectures were analyzed: unimodal, multimodal, and multi-image models. The best unimodal model only misclassified one pathological eye as healthy, specifically categorizing one healthy eye as RVO. This is particularly significant and promising for disease diagnosis, since false positives should be minimized. Additionally, this model misclassified DME eyes as RVO and vice versa, indicating that these two classes have similar features, which aligns with the clinical challenge of distinguishing between DME and RVO.

The best multimodal model, which included additional input about the patient’s diabetes diagnosis, demonstrated the value of this information. This model correctly classified all DME images and showed overall better results for every metric presented, underscoring the benefits of using diabetes-related information. Furthermore, this model did not produce any false positives but still had difficulty differentiating RVO from DME, misclassifying 16 eyes with RVO as DME. The integration of the diabetes diagnosis and IR imaging increased the model accuracy. This approach can potentially be extended to other retinal diseases and integrated into clinical workflows.

The unimodal model misclassified three normal eyes as DME, and these patients did not have diabetes. In the multimodal model, this error would not occur since a non-diabetic patient’s entry would never be classified as DME. Only eyes from diabetic patients were classified as DME in the multimodal model, including the misclassified ones. The higher number of false DME positives indicates that the diabetes information introduced some bias, causing the model to classify more eyes as DME.

The multi-image model achieved the best results among the three approaches in the three-class analysis. This approach had better differentiation between the DME and RVO classes, with only ten misclassified eyes between these two classes. All the evaluation metrics were higher for this approach, highlighting the positive impact of including an IR image as an input. The IR image also helped mitigate the bias mentioned earlier. These results highlight the value of multi-modal approaches in enhancing classification accuracy.

The four-class model showed better results than the unimodal and multimodal models in the three-class problem. However, its performance fell short of the multi-image model in the three-class problem, indicating that the added class increased the complexity of the task. In this model, all eyes classified as DME had diabetes or lacked diabetes diagnostic information, demonstrating that the model understood that only diabetic patients can have DME. One DME eye was misclassified as normal, showing that the model does not automatically classify all diabetes-related images as DME. Besides this false negative, the model also predicted two eyes with other pathologies as normal. Regarding the distinction between DME and RVO, the model misclassified eleven RVO eyes as DME.

Despite the increased complexity of the four-class model, which includes a class for other pathologies, the method demonstrates promising clinical applicability. The inclusion of this additional class is crucial, as it aligns better with real-world scenarios where multiple pathologies may be present. While the ‘Other’ class does not provide a specific diagnosis, it prevents the model from misclassifying non-edematous retinal pathologies as healthy or as edema secondary to RVO or DME. This design ensures that the model serves as a robust preliminary screening tool, prompting clinicians to conduct further evaluations for conditions beyond the scope of the current classification framework.

In the testing set, images of eyes with diabetes appeared in all classes, as shown in Table 4, and these were correctly classified, indicating that the model did not classify all eyes from diabetic patients as DME.

Class-wise metrics were calculated to properly compare the proposed work with existing studies, since state-of-the-art models have a different number of classes than those used in this work. Table 8 presents the class-wise and overall metric results for the proposed models and their comparison to the state of the art. The state-of-the-art models listed have more classes than those in Table 8, but the comparison focuses on DME and RVO. Section 1 details the specific classes used in each study.

The overall metrics were calculated based on the weighted average of all classes. For better comparison with other published works, the metrics from those works were inferred from their confusion matrices. Consequently, the results presented in their papers might differ from those reported here due to different average metric calculations. The RVO class-wise metric for the reviewed state-of-the-art models was based on the mean results for CRVO and BRVO. The proposed model achieves higher overall accuracy than those by Abitbol et al. [13], Pin et al. [17], and Kulyabin et al. [19], falling short only of Khan et al. [18], who reported an accuracy of 99.17%. However, the proposed work used a dataset with more patients and images, and the use of a class for other pathologies increased the classification task’s complexity.

Regarding the class-wise metrics, the proposed model achieved a higher accuracy and recall for DME and RVO than all the works, except for Khan et al. [18], which had higher accuracy and recall for RVO only. The model uses additional inputs compared to the current literature, accounting for an enriching innovative step that justifies the better results achieved. Regardless of this additional input, the unimodal model application still achieves better results than the current state of the art, with the exception of Khan et al. [18]. This comparison demonstrates the proposed model’s strong performance relative to the existing literature. Only works that used some OCT scans of each patient are part of the current literature state-of-the-art comparison. Works that used the whole volume of a patient were excluded.

The proposed model offers a clinically applicable approach to distinguishing between RVO and DME and enhances the need to include multimodality imaging and clinical information in the training of AI algorithms. The inclusion of an additional class for other pathologies enhances clinical applicability and differentiates this work. The model’s efficiency is evidenced by its accurate differentiation of eyes in the dataset.

Even though DME or BRVO-related edema can manifest outside the central foveal scans, the dataset used focuses on the five scans nearest the fovea. This decision was motivated by the fact that the central region of the retina is critical for visual acuity, and diseases that impact these regions often require treatment. Macular diseases occurring outside the central region frequently do not necessitate immediate treatment. Therefore, by focusing on the central scans, the model is useful as a screening tool for retinal diseases that impact visual outcomes, confirming disease presence within the central foveal and parafoveal regions. This targeted approach ensures that only patients with treatable conditions affecting vision are flagged for further evaluation, optimizing the screening process and minimizing over-referrals.

However, the work has some limitations. The use of images from a single device may reduce the model’s clinical applicability to other devices, and in the future, it would be important to test the model on images from other devices and consider the incorporation of images from other devices on model training. The model can tell if a patient is healthy or classify them with DME, RVO, or other pathology. In case of a classification with other pathology, the clinicians still need to provide a more accurate diagnosis and identify the pathology. Thus, in future works, it will be necessary to increase the number of classes and specify more retinal diseases.

## 5. Conclusions

This study presents a novel method to distinguish edema secondary to RVO and DME using a combination of retinal OCT images, diabetes diagnostic information, and IR images. The results indicate that including diabetes diagnostic information and IR images alongside OCT scans significantly improves the model’s performance, particularly in distinguishing between edema secondary to RVO and DME, which have similar visual characteristics that pose a diagnostic challenge for ophthalmologists.

The findings suggest that the proposed method could significantly impact clinical practice by practice by enabling earlier and more accurate differentiation between edema secondary to RVO and DME, leading to timely treatment and improved patient outcomes. However, relying only on images from a single device may limit generalizability, and future work should validate the approach with data from diverse imaging systems.

In summary, this method advances the field of retinal image classification and demonstrates the potential of AI to transform patient care, improving the accessibility and efficiency of healthcare.

## Figures and Tables

**Table 1:** Number of images and patients from the Kermany dataset [16].

	CNV	Drusen	DME	Normal	Total
Images—Original	37,206	8617	11,349	51,140	108,312
Images—After removing duplicates	31,838	8118	11,171	50,317	101,444
Patients	968	875	880	3605	6328

CNV—choroid neo-vascularization; DME—diabetic macular edema.

**Table 2:** HSJ dataset distribution.

	Normal	DME	RVO	Other
B-scans, n	1040	1090	1035	1000
Volumes, n	208	218	207	200
IR, n	208	218	207	200
Patients, n	204	202	205	155
Age (years), mean (SD)	56.16 (17.14)	65.14 (11.41)	70.13 (11.60)	59.93 (20.77)
Gender, n (%)				
Male	85 (41.67)	120 (55.05)	104 (50.24)	101 (50.50)
Female	119 (58.33)	98 (44.96)	103 (49.76)	99 (49.50)
Eye, n (%)				
Right	105 (51.47)	113 (51.83)	108 (52.17)	103 (51.50)
Left	99 (48.53)	105 (48.17)	99 (47.82)	97 (48.5)
Patients with Diabetes, n (%)	36 (17.65)	202 (100.00)	48 (23.41)	29 (18.71)

DME—diabetic macular edema; RVO—retinal vein occlusion; SD—standard deviation; n—number.

**Table 3:** Split of Kermany dataset into training, validation, and testing sets.

	CNV	Drusen	DME	Normal
Training and Validation Set	31,588	7868	10,921	50,067
Testing Set	250	250	250	250

CNV—choroid neo-vascularization; DME—diabetic macular edema.

**Table 4:** Number of images per class of the training, validation, and testing sets.

	Normal			DME			RVO			Other		
	D+	D-	D?	D+	D-	D?	D+	D-	D?	D+	D-	D?
Training Set	145	640	5	840	0	0	180	605	0	145	575	30
Validation Set	15	110	0	125	0	0	15	110	0	20	95	10
Testing Set	20	105	0	125	0	0	45	75	5	20	105	0

DME—diabetic macular edema; RVO—retinal vein occlusion; D−—without diabetes; D+—with diabetes; D?—without information.

**Table 5:** Hyperparameter tuning.

Parameter	Values			
α	0	0.001		0.01
Data augmentation	Gaussian Blur	Horizontal Flip		Horizontal Flip + Gaussian Blur
Dropout	0.5	0.6	0.7	0.8
$T_{\max}$	0	5		10
Activation function	ReLU	LeakyReLU	Tanh	ELU
TL	Feature extractor	Fine-tuning		Fine-tuning from scratch
Initial learning rate	0.0001		0.00001	
Neurons on the hidden layer	256	512		1024
Batch size	16	32		64

α—regularisation parameter; $T_{max}$—maximum number of iterations for cosine annealing schedule; ReLU—rectified linear unit; TL—transfer learning.

**Table 6:** Comparison of results on Kermany dataset.

	F_0.5_ Score	Precision	Recall	Accuracy
Li et al. [11]	98.61	98.62	98.60	98.60
Our Model	85.52	86.17	85.20	85.20

**Table 7:** Average and best metrics for the models trained with the three architectures proposed.

	Unimodal		Multimodal		Multi-Image	
	Average	Max	Average	Max	Average	Max
F_0.5_ (%)	88.49 ± 0.52	92.32	91.27 ± 0.48	95.04	93.30 ± 0.44	96.71
A (%)	88.23 ± 0.57	92.26	90.81 ± 0.50	94.67	92.92 ± 0.52	96.53
P (%)	88.67 ± 0.49	92.34	91.70 ± 0.41	95.40	93.61 ± 0.46	96.86
R (%)	88.23 ± 0.57	92.26	90.81 ± 0.50	94.67	92.92 ± 0.52	96.53
AUROC (%)	96.66 ± 0.21	97.48	97.25 ± 0.23	99.06	97.64 ± 0.25	97.52

F_0.5_—F_0.5_ score; A—accuracy; P—precision; R—recall; AUROC—area under the receiver operating characteristic curve.

**Table 8:** Class-wise and overall metrics of the published and proposed works.

Model	Dataset	Images	Class	F_0.5_ Score	Accuracy	AUROC	Precision	Recall
Fundus Images:								
Abitbol et al. [13]	Private	224	DR	76.12	67.69	90.5	78.57	67.69
			RVO	71.43	78.72	91.2	69.81	78.72
			No	72.55	67.27	88.5	74.00	67.27
			Overall		88.39		76.37	71.11
OCT Images:								
Pin et al. [17]	Private	1999	DME	88.39	99.00	-	86.08	99.00
			RVO	91.21	78.97	-	94.90	78.97
			Overall	91.75	91.69	-	92.05	91.69
Khan et al. [18]	Private	2998	DME	99.20	98.68	-	99.34	98.68
			RVO	98.97	99.43	-	98.88	99.43
			Overall	99.17	99.17	100	99.17	99.17
Kulyabin et al. [19]	OCTDL [20]	1618	DME	88.65	86.21	-	89.29	86.21
			RVO	72.82	78.95	-	71.43	78.95
			No	88.93	91.38	-	88.33	91.38
			Overall	92.18	92.10	98.00	92.24	92.10
Proposed:								
Unimodal 3 classes	HSJ	3165	DME	97.26	98.46	96.97	98.46	99.90
			RVO	96.92	96.92	96.92	96.92	99.80
			No	99.69	98.46	100	98.46	100
			Overall	97.96	97.95	99.87	97.96	97.95
Multi-image 3 classes	HSJ	3165	DME	100	100	100	100	100
			RVO	99.37	96.92	99.30	100	96.92
			No	97.60	100	99.80	97.62	100
			Overall	98.99	98.97	99.72	99.00	98.97
Multi-image 4 classes	HSJ	4165	DME	91.04	99.20	97.60	89.21	99.20
			RVO	94.64	90.40	94.53	95.76	90.40
			No	97.42	96.80	98.00	97.58	96.80
			Other	96.72	98.40	97.07	99.16	94.40
			Overall	95.38	95.20	99.59	95.43	95.20

DME—diabetic macular edema; RVO—retinal vein occlusion; DR—diabetic retinopathy; No—normal; AUROC—area under the receiver operating characteristic curve.

## Data Availability

The datasets presented in this article are not readily available because the data are part of an ongoing study.
